# Effects of Nanoscale V-Shaped Pits on GaN-Based Light Emitting Diodes

**DOI:** 10.3390/ma10020113

**Published:** 2017-01-28

**Authors:** Shuo-Wei Chen, Heng Li, Chia-Jui Chang, Tien-Chang Lu

**Affiliations:** 1 Department of Photonics, National Chiao Tung University, Hsinchu 300, Taiwan; yweiweiwei@gmail.com (S.-W.C.); joe0ee9@yahoo.com.tw (H.L.); blue12480315@gmail.com (C.-J.C.); 2 Epistar Corporation, Hsinchu 300, Taiwan

**Keywords:** light emitting diodes (LEDs), GaN, multiple quantum wells (MQWs)

## Abstract

This paper reviews the formation of nanoscale V-shaped pits on GaN-based light emitting diodes (LEDs) grown by the metal organic chemical vapor deposition (MOCVD) system and studies the effect of V-shaped pits on quantum efficiency. Since V-pits could provide potential barriers around threading dislocations to lessen non-radiative recombinations in such a high defect environment. In our study, multiple InGaN/GaN quantum well samples with different emission wavelengths of 380, 420, 460, and 500 nm were grown, each with different nanoscale V-shaped pits of three diameters for 150, 200, and 250 nm, respectively. It was found that the multiple quantum well (MQW) sample with larger V-pits had a lower pit density, but a relatively larger total V-pits defected area. The optimum diameter of V-pits showing the highest quantum efficiency from the MQW sample depended on the emission wavelength. MQW samples with wavelengths of 380 and 500 nm exhibited the best internal quantum efficiency (IQE) performance at the smallest V-pits area; however, the best performance for MQW samples with wavelength around 420 and 460 nm occurred when large V-pit areas were presented. Photoluminescence (PL) peak shifts and Raman shifts can provide a relationship between quantum-confined Stark effect (QCSE) and IQE, as well as a comparison between strain and IQE. The results obtained in this phenomenological study shall provide a useful guide line in making high-performance GaN-based LEDs with wide emission spectra.

## 1. Introduction

Solid state lighting of GaN-based light emitting diodes (LEDs) has already become the most significant illumination technology in recent years because it has several advantages, including high conversion efficiency, long lifetime, and ecologically friendliness [1,2,3,4,5,6]. GaN-based LED structures are typically made on (0001) c-plane sapphire substrates due to the lack of natural GaN substrates [7,8,9]. A large amount of threading dislocations (TDs) may be caused by the interfacial energy difference from lattice constant and thermal expansion coefficient mismatch between sapphire substrates and GaN [10,11]. Intensive non-radiative recombination centers resulting from TDs severely reduce the quantum efficiency of the light emitting devices [12,13,14,15,16]. It should be noted that V-shaped pits are generally observed in the InGaN/GaN multiple quantum wells (MQWs) along the TDs, and they typically have a shape with open hexagonal, inverted pyramids with (10-11) faceted sidewalls [17,18,19]. Previous studies have claimed that the pre-strained layers or superlattice (SLS) embedded under MQWs in the LED structure could relax strain within layers. However, more SLS pairs would accumulate the strain energy, and the partial strain relaxation would also trigger the formation of a V-pit, and an optimized V-pit size is about 200–250 nm with the opening angle of 60° [20,21,22]. Several reports showed that these V-shaped pits can perform positively in InGaN-based LEDs, such as in the suppression of non-radiative recombination and contributing to a significant increase in the light emission efficiency [23,24].

Recently, electrical and optical properties have been improved by carefully control and engineering of the interfacial strain energy and dislocations so that high efficiency of blue InGaN-based LED can be promoted [25,26]. Tomiya et al. used high-angle annular dark field scanning transmission electron microscopy and atom probe tomography to confirm the existence of thin quantum wells in the slope region of V-pits, and the thicknesses and In concentrations were much lower than those of the flat region. This demonstrated that the threading dislocations in the V-shaped pits act as energy barriers for the lateral transport of charge [27]. In 2014, Kim et al. discussed the influence of V-pits and their energy barrier originating from its facets of (1011) planes, and it revealed that higher V-pit energy barrier heights in InGaN QWs more efficiently suppress the non-radiative recombination at TDs, thus enhancing the internal quantum efficiency (IQE) [28]. Chang et al. applied superlattice layers to manipulate the nanoscale V-pits and obtain the optimum V-pits size to achieve high-efficiency blue wavelength InGaN/GaN LEDs [22].

To study the effect of V-pits on efficiency of InGaN MQWs more systematically, in the present study, three sizes of V-shaped pits (150, 200, and 250 nm) with four wavelengths of MQW samples (380, 420, 460, and 500 nm) were fabricated on 4-inch patterned sapphire substrate (PSS) by metal organic chemical vapor deposition (MOCVD). The structural and optical characteristics of the as-grown wafers were investigated, and the suitable size of V-shaped pits for each wavelength was therefore discussed.

## 2. Experiments

**Figure 1:**
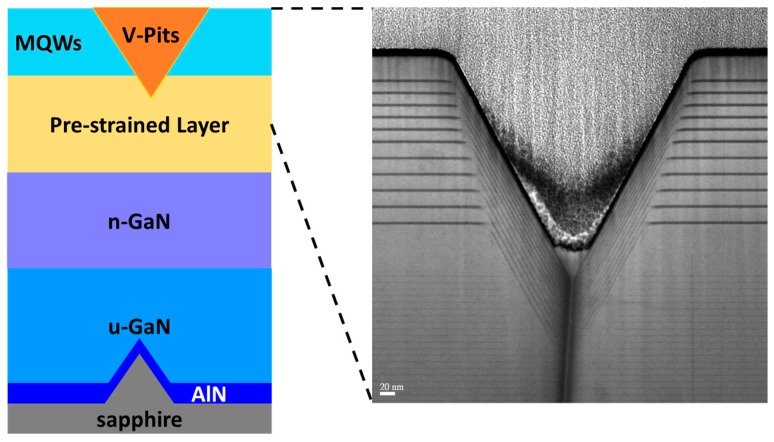
Schematic diagram of structure with an ex-situ physical vapor deposition (PVD) AlN nucleation layer. The diameter, space, and height of the cone on the patterned sapphire substrate (PSS) were 2.8, 0.2, and 1.8 μm, respectively. The enlarged image shows the nanoscale V-shaped pit taken by the TEM. MQW: multiple quantum wells; uGaN: unintentionally-doped GaN.

The InGaN-based LED structures were grown on the 4-inch (0001) PSS by MOCVD. Trimethylgallium (TMGa), trimethylaluminium (TMAl), trimethylindium (TMIn), and ammonia (NH_3_) were used as precursors of chemical reactions for Ga, Al, In, and N sources, respectively. N-type and p-type doping sources were silane (SiH_4_) and bicyclopentadienyl magnesium (Cp_2_Mg), respectively. The schematic structure of the epitaxial layer and a TEM image of V-pit are shown in Figure 1. The layer structure includes an ex-situ 25-nm-thick physical vapor deposition (PVD) AlN nucleation layer deposited first on the PSS. This ex-situ AlN layer has been proved to offer better crystal quality of the following GaN than the conventional low-temperature GaN nucleation layer [29]. After that, InGaN-based LED structures consisted of a 4-μm-thick unintentionally-doped GaN (u-GaN) layer, a 3-μm-thick n-type GaN layer (n-doping = 1 × 10^19^ cm^−3^), a pre-strained layer consisted of several pairs of GaN/InGaN structure which is with a 25-nm-thick n-doping GaN and a 2.5-nm-thick un-doped InGaN grown beneath the MQWs to form V-shaped pits with different sizes. The thickness of pre-strained layer was varied to precisely control the formation of V-pits. Several pairs of InGaN/GaN MQWs with a 3-nm-thick un-doped well and a 12-nm-thick n-doping barriers as active regions, and the wavelengths of 380, 420, 460, and 500 nm were subsequently grown, and the determination of the indium composition of MQWs was controlled by the growth temperature of well layers. After the samples were grown, the diameter of V-shaped pits of samples was analyzed by scanning electron microscope (SEM). The optical properties of the InGaN/GaN MQWs were investigated by cathodoluminescence (CL) and measuring the power-dependent photoluminescence (PL) at low and room temperature. Two different wavelengths of pumping source were used to measure the power dependent PL spectrum and determine the power-dependent internal quantum efficiency (IQE), and both of them were generated by mode-locked Ti:sapphire pulse laser (Mira 900) with the pulse width of 200 fs and repetition rate of 76 MHz. The samples with 460 and 500 nm emission wavelength were bumped by 400 nm pulse laser, second harmonic generation of Ti:sapphire laser, and the samples with 380 and 420 nm emission wavelength were pumped by a 266 nm pulse laser, third harmonic generation of a Ti:sapphire laser. The power-dependent spectrum of the green LED sample was measured by the micro-PL system, and increased maximum power density could be measured by reducing the pumping spot size. A Mitutoyo objective, 100×, NA0.5 was used to focus the laser light onto the sample, and achieved a spot size of 3.5 μm. In addition, all the pumping powers are average power values. The quantum efficiency was defined as the photon numbers emitted from the sample divided by the excitation photon numbers. Here, the IQE was evaluated by the ratio between the highest quantum efficiency obtained at room temperature and low temperature. Quantum efficiency was computed by dividing integrated PL intensities by the pumping power for each pump power, and normalized by maximum at low temperature for each sample to get the IQE value. The strain of the thin film structure was investigated by measuring Raman spectra. The excitation source was a 532 nm laser light, and the scattered light was collected by a 100× NA0.7 objective lens. Since the phonon frequency shift was linearly proportional to both the biaxial and uniaxial strain in GaN [30,31], the local strain can be evaluated by analyzing the Raman peak position. The quantum efficiency was defined as the photon numbers emitted from the sample divided by the excitation photon numbers [32], and the IQE was defined by the ratio between the highest quantum efficiency obtained at room temperature and low temperature [33]. 

## 3. Results and Discussion

### 3.1. Diameter and Density of V-Shaped Pits

**Figure 2:**
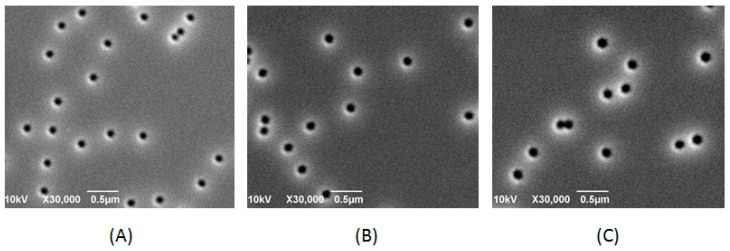
SEM top view images of MQW layers grown on PSS with a pre-strained layer of various thickness grown beneath the MQWs to form V-shaped pits with different sizes. (**A**) 150 nm V-pits; (**B**) 200 nm V-pits; and (**C**) 250 nm V-pits, respectively.

**Figure 3:**
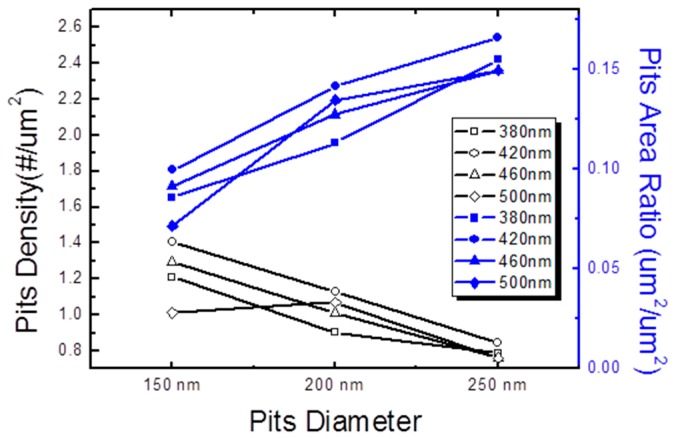
Pits density and pits area ratio as a function of pits diameter.

Figure 2A–C show the surface morphology of V-shaped pits with different pre-strained layer thicknesses. The diameters of V-pits on various pairs of pre-strained layers were 150, 200, and 250 nm, respectively. From former research about the formation of V-shaped pits [22,34,35,36,37], we can understand that the beginning of these V-pits started from TDs which were originated from the c-plane region because of the lattice constant difference between PSS and GaN layers, and then extended along the direction of the c axis. At the end of threading edge dislocation, the V-shaped pits were generated [18,22,38,39]. After calculation of the density of V-pits, we can have the relationship between V-pits density and diameter, as shown in Figure 3. The pits density of samples with 150, 200, and 250 nm V-shaped pits diameters were around 1.3, 1.0 and 0.8/μm^2^, respectively. There is a correlation between pits density and pits diameter. The larger the pits diameter is, the lower the pits density is. Indeed, during the growth of pre-strained release layers, some V-shaped pits might be covered by later GaN/InGaN layers. However, when we converted it to the ratio of pits area (as shown in Figure 3), an obvious linear relationship between the ratio of pits area and pits diameter was revealed. Larger pits have a larger ratio of area regardless of the emission wavelength. This could lead to a situation where the sample with larger V-pits diameter might have a smaller area for radiative carrier recombination, because V-pits area centering the dislocations shall be dominated by the non-radiative recombination process.

### 3.2. SEM and CL Images

**Figure 4:**
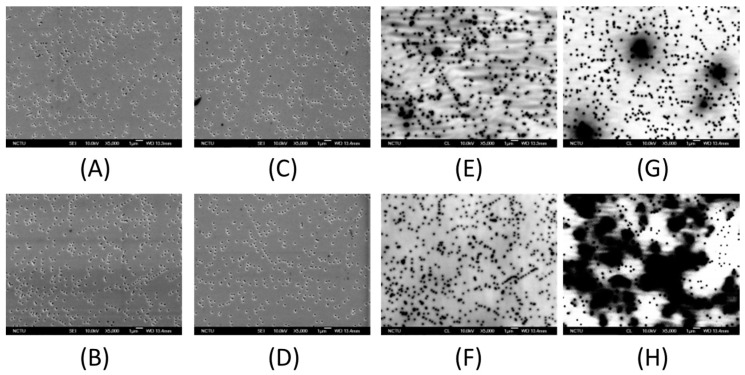
The SEM image MQWs with 250 nm diameter V-pits at wavelength of (**A**) 380 nm; (**B**) 420 nm; (**C**) 460 nm; and (**D**) 500 nm. The plane-view monochromatic cathodoluminescence (CL) image of MQWs with 250 nm diameter V-pits at wavelength of (**E**) 380 nm; (**F**) 420 nm; (**G**) 460 nm; and (**H**) 500 nm.

The extended SEM top view images of V-shaped pits samples at wavelength of 380, 420, 460, and 500 nm are shown in Figure 4A–D. The samples all have pit size of 250 nm. Nevertheless, these figures clearly show that the samples with larger V-pits have a lower pits density. On the other hand, Figure 4E–H show the monochromatic CL-images of V-shaped pits samples at wavelengths of 380, 420, 460, and 500 nm, respectively. The positions of V-pits are related to the dark spots shown in Figure 4A–D, which represent the non-radiative recombination centers. Except for those small dark spots caused by V-pits, some shadow regions existed in the samples with longer wavelength. This could be due to the appearance of high indium composition clusters at longer wavelengths such as 460 or 500 nm, which may also result in non-uniform radiative recombination. When it comes to the region with no V-pits and shadow regions in the CL images of Figure 4E–H, the one at 460 nm wavelength showed the strongest emission intensity compared to those at 420 and 380 nm, which means that 250 nm-diameter V-pits could provide a better light emission performance with 460 nm wavelength. However, sample at 500 nm wavelength possessed a too-large V-pits area and indium shadow region, which could cause poor emission intensity. Further optical investigation was carried out and is discussed in the following.

### 3.3. IQE with Different Diameters of V-Shaped Pits at Various Wavelengths

**Figure 5:**
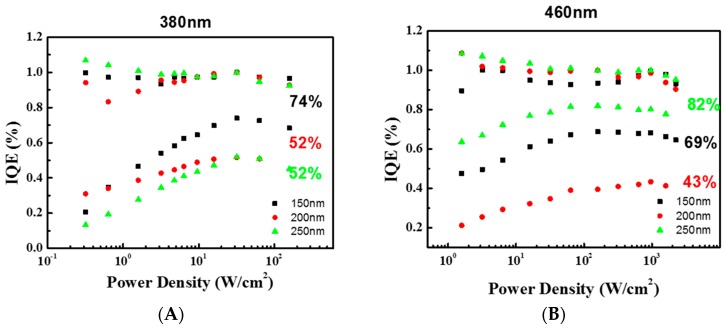

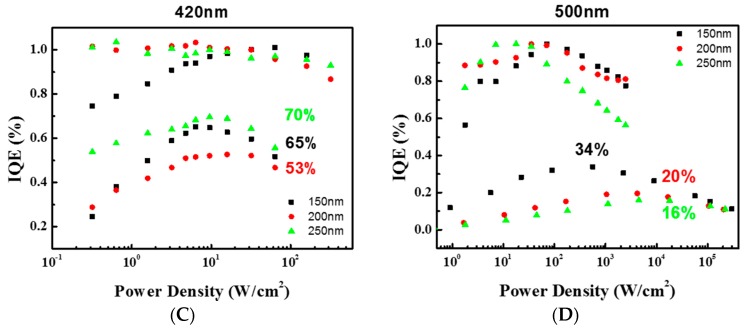
The normalized internal quantum efficiency (IQE) values of MQWs as a function of power density at 12 and 300 K with varying V-pits diameters of 150, 200, and 250 nm, respectively, at wavelength of (**A**) 380 nm; (**B**) 420 nm; (**C**) 460 nm; and (**D**) 500 nm.

All the samples were analyzed by the power-dependent PL at 12 and 300 K. The measured quantum efficiency data were normalized by the maxima value at 12 K for all samples as IQE value of 100% and the peak value at 300 K was defined as the room temperature IQE value as shown in Figure 5A–D. As the power density was low, the IQE showed an obvious increasing trend as the power density increased. This could be due to the screening of the non-radiative recombination centers caused by the point defect. As the power density increased, the injected carriers would gradually compensate the point defects, leading to an increase in IQE value. This phenomenon was more significant when the measurement temperature was at room temperature, because the thermal agitation could provide energy to carriers to be trapped by the point defects. On the other hand, when the power density was very high, the IQE showed an obvious decreasing trend, which was identified as a notorious efficiency droop effect. As a result, a highest IQE value would be observed at specific pumping power density. Numerous reports have debated on the roots of droop, because the postponed onset of droop effect could increase the highest IQE value to be observed [40,41]. Here, we systematically showed the effect of different V-pits on the highest IQE values on different emission wavelength samples.

**Figure 6:**
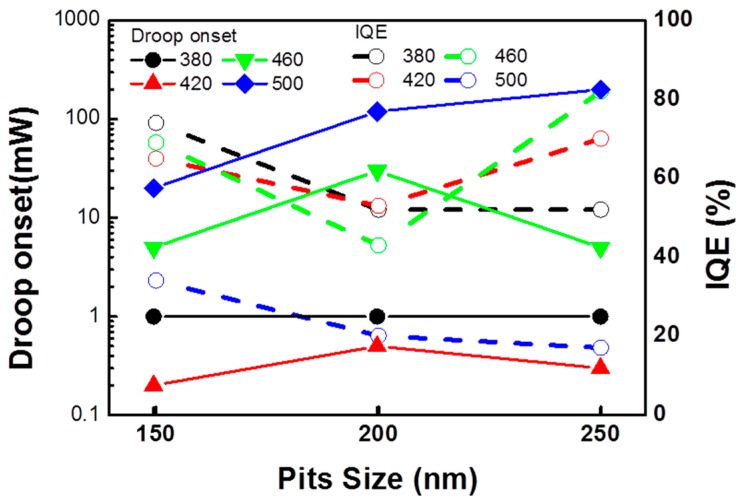
The droop onset and IQE values as a function of V-shaped pits diameter for samples with different emission wavelength of 380, 420, 460, and 500 nm, respectively.

Figure 6 shows the droop onset and IQE values as a function of V-shaped pits diameter for samples with different emission wavelength. It can be divided into two groups: one contains samples emitted 420 and 460 nm, and the other contains samples emitted 380 and 500 nm. In the group of 420 nm/460 nm, the worst IQE value of around 50% was found at V-pits diameter of 200 nm, which could be due to the fact that the highest percentage of carriers are trapped by the TDs. In addition, the IQE value at 460 nm wavelength was even lower, which was because of the shadow region caused by the indium cluster. On the other hand, in the group of 380 nm/500 nm, the IQE value decreased with larger V-pits diameter. For the case of 380 nm wavelength, the indium distribution is relatively uniform in the CL images of Figure 4E–H, carriers in this sample may need more area without V-pits to recombine. So, sample with the largest area without V-pits—the 150 nm V-pits sample—is the one with the highest IQE. However, the situation in the sample of 500 nm wavelength was different. Because of the large occupation of shadow region and V-pits in the large pit samples, a lower IQE value was observed. Besides, in terms of the IQE value among all the samples, that with 250 nm-diameter V-pits and 460 nm emission wavelength showed the best IQE value because the potential barrier at the side walls of V-pits could provide the best energy height for blocking the carriers trapped into the non-radiative recombination centers along with the TDs. For IQE values, 420 and 380 nm wavelength samples came in the 2nd and 3rd position, respectively, which showed a decreasing trend of potential barrier height around these V-pits. The one at 500 nm wavelength exhibited the worst IQE value, which could be due to the obsoleted effect of V-pits. The reason that these V-pits no longer had an effect on the 500 nm sample might result from the poor material quality in high indium content MQWs. This trend can match the emission intensity of CL results previously discussed in Figure 4E–H. Furthermore, an opposite trend could be observed between droop onset and IQE. In the previous research, lower efficiency droop onset can be attributed to stronger carrier localization, caused by both content fluctuations and QW width fluctuations [42]. Besides, the fluctuation and higher localization effect can prevent the carrier diffusion to the extended defects and contribute to a mitigation of the droop effect [43].

**Figure 7:**
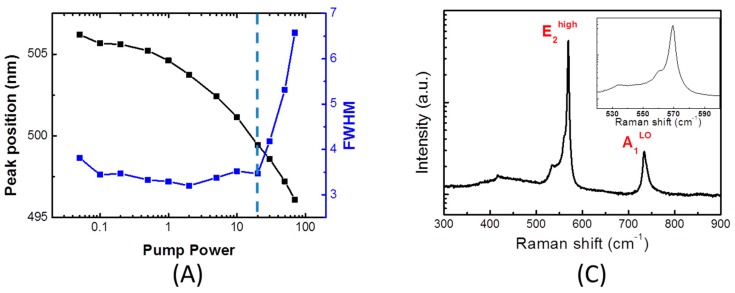

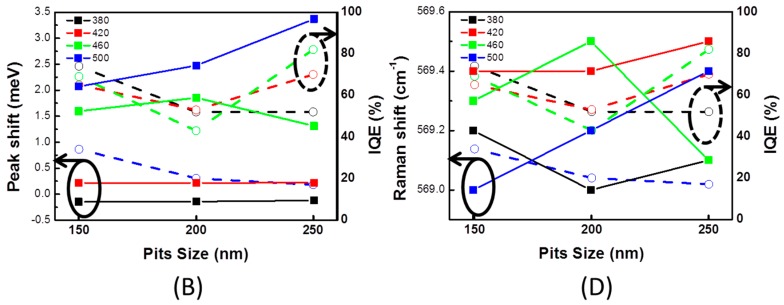
(**A**) Peak position and full width at half maximum (FWHM) as a function of pump power (dashed line is the bound of QCSE screening and Burstein–Moss effect); (**B**) Comparison chart of peak shift and IQE; (**C**) Raman spectrum of 460 nm sample with 250 nm V-shaped pits. Inset shows a zoomed-in view of the E_2_^high^ peak. (**D**) Comparison chart of Raman shift and IQE.

Figure 7A shows the PL emission peak and spectra full width at half maximum (FWHM) of sample as a function of excitation power. At the lower excitation power, the FWHM of PL emission peak decreased with the blue-shift of PL emission peak, because the QCSE was gradually screened and the MQWs became flatter with increasing carrier density. At the higher excitation power, the QCSE was completely screened, and the Burstein–Moss effect started to dominate the blue-shift of the PL emission peak. Meanwhile, many carriers were pushed to higher energy states because all states close to the conduction band were populated, so the FWHM of PL emission peak increased. In this study, QCSE-determined range of lower excitation power is discussed. Figure 7B shows the comparison chart of peak shift and IQE as a function of V-pits diameters. It can be observed that the peak shifts correspond to the wavelength, which can also be defined as indium composition of MQW. In terms of 380 and 420 nm samples, they almost keep at the same value of peak shift, which means the QCSE is relatively low in samples with shorter wavelength [44], and the IQE of 380 and 420 nm are not affected by the QCSE within samples. However, the other two samples of 460 and 500 nm show obvious QCSE, and are inversely proportional to IQE. On the other hand, Figure 7C,D show Raman spectrum of 460 nm sample with 250 nm V-shaped pits and comparison chart of Raman shift and IQE, respectively. All samples are under compressive strain, because the E_2_^high^ Raman peak was higher than that of unstrained GaN bulk occurring at 567.6 cm^−1^ [30,31]. The result can also be separated to two groups. One is 380 nm/420 nm, and the Raman shifts are proportional to IQE. However, the relationship of Raman shifts and IQE in the 460 nm/500 nm group are the contrary.

Finally, an interesting relationship of all the analysis results can be used as a method to estimate the relative IQE at the same wavelength. Table 1 shows the products of area ratio without V-pits, the reciprocal of PL peak shift, and the reciprocal of Raman shift compared to the measured IQE values. For the samples of the same wavelength, the products have the same trend with IQE value. As a matter of fact, the area ratio without V-pits and the reciprocal of PL peak shift determine the IQE values, indicating that less V-pits area and smaller PL peak-shift would lead to a better IQE performance.

## 4. Conclusions

We have systematically demonstrated the controllability of nanoscale V-pits in InGaN/GaN MQWs by using MOCVD. The pits density of samples with 150, 200, and 250 nm V-shaped pits diameters gradually decreased. However, when it was converted to area, we can have the largest total V-pits area with 250 nm-diameter V-pits and smallest total V-pits area with 150 nm-diameter V-pits sample. CL measurement results showed that dark spots matched well to V-pits positions which behaved as non-radiative recombination centers. In addition, samples with longer wavelength were found to be covered with shadow regions in CL images, which may be due to the high indium composition cluster. Moreover, the 460 nm wavelength sample showed the strongest emission intensity, indicating that it had fewer carriers trapped by TDs and the most suitable area to execute radiative recombination. The 380 and 500 nm wavelength samples had the highest IQE value at the smallest V-pits area, indicating that carriers in these two samples required larger area to recombine. On the other hand, PL peak shifts reveal that the QCSE is relatively low in samples with shorter wavelength of 380 and 420 nm, and the other samples of 460 and 500 nm show that their IQE are inversely proportional to QCSE. Finally, an interesting result that using the products of area ratio without V-pits, the reciprocal of PL peak shift and the reciprocal of Raman shift can approximately estimate the relative IQE at the same wavelength. Based on this systematic research, we can understand that the best IQE performance may not occur at the same V-pits diameter or area. The inter-relationships among structure, wavelength, V-shaped pits area, QCSE, and strain should be considered. These results could provide a general guide to guarantee high performance InGaN light emitting devices applied in wide emission wavelength bands.

## Figures and Tables

**Table 1:** The products of area ratio without V-pits, the reciprocal of photoluminescence (PL) peak shift and the reciprocal of Raman shift compared to the measured IQE values.

Wavelength (nm)	Pits Size (nm)	(A) Area Ratio without V-Pits	(B) Reciprocal of PL Peak Shift (1/nm)	(C) Reciprocal of Raman Shift (1/cm^−1^)	(A)*(B)*(C)	IQE
380	150	0.91	5.00	0.1757%	0.803%	74%
	200	0.89	5.00	0.1757%	0.779%	52%
	250	0.85	5.00	0.1757%	0.743%	52%
420	150	0.90	4.00	0.1756%	0.633%	65%
	200	0.86	3.85	0.1756%	0.580%	53%
	250	0.83	4.35	0.1756%	0.637%	70%
460	150	0.91	0.63	0.1757%	0.100%	69%
	200	0.87	0.57	0.1756%	0.088%	43%
	250	0.85	0.77	0.1757%	0.115%	82%
500	150	0.93	0.48	0.1757%	0.078%	34%
	200	0.87	0.40	0.1757%	0.061%	20%
	250	0.851	0.30	0.1756%	0.045%	17%
